# In vivo CAR-T cell engineering: concept, research progress, potential challenges and enhancement strategies

**DOI:** 10.1186/s40164-025-00725-5

**Published:** 2025-11-18

**Authors:** Yizhao Chen, Qianling Xin, Jiaqi Qiu, Mengjuan Zhu, Zixuan Li, Ji Qiu, Jiajie Tu, Ruilin Li

**Affiliations:** 1 Department of Pharmacy, The Third Affiliated Hospital of Anhui Medical University, Hefei First People’s Hospital, 390# Huaihe Road, Luyang District, Hefei, China; 2 Anhui Women and Children’s Medical Center, Hefei Maternal and Child Health Hospital, Hefei, China; 3 Key Laboratory of Anti-Inflammatory and Immune Medicine, Ministry of Education, Anhui Collaborative Innovation Center of Anti-Inflammatory and Immune Medicine, Institute of Clinical Pharmacology, School of Pharmacy, Anhui Medical University, 81# Meishan Road, Shushan District, Hefei, China

**Keywords:** In vivo CAR-T, Adoptive therapy, Cancer, Gene editing

## Abstract

After decades of development and accumulation, chimeric antigen receptor (CAR)-T therapy has become a revolutionary immunotherapy method, which has triggered changes in treatment methods and concepts in the fields of cancer, autoimmune disorders, infection, fibrosis and other diseases. With the continuous expansion of indications and potential application fields, adoptive CAR-T therapy products are difficult to meet the expanding market demand and provide equal access to treatment due to their technical complexity and substantial production costs. These factors drive the development and practice of novel technologies, in this context, in vivo CAR-T therapy has been proposed: the in vivo or in situ programming of CAR-T cells to eliminate pathological cells through the delivery of CAR genes in vivo by viruses or engineered nanoparticles. This new technology pathway simplifies the manufacturing and therapeutic procedures, reduces treatment costs, and improves patient accessibility, which has excellent potential for clinical application. This article reviews recent advances in in vivo CAR-T therapy, compares the advantages and characteristics of this approach with traditional adoptive therapy, discusses the therapeutic risks and related challenges of in vivo CAR-T therapy, and emphasizes the guiding significance of adoptive therapy-based enhancement strategies for the development of in vivo CAR-T therapy.

## Introduction

The emergence and development of chimeric antigen receptor (CAR) immune cell therapy has brought new hope for the management of cancer, autoimmune diseases and other refractory diseases [1, 2]. Through the binding of synthetic CAR molecules to target antigens, the corresponding functions of various immune cells were activated, enabling the elimination of cancer cells or other pathogenic cells to alleviate or improve disease symptoms [3]. At present, the main therapies developed include CAR-T, CAR-NK, CAR-Macrophage (CAR-M) and CAR-Treg, which have led to significant transformations in treatment methods and concepts in the fields of cancer, autoimmune diseases, aging, and organ transplantation [4–6].

Since achieving breakthroughs in clinical studies in 2011, CAR-T therapy has remained the most mature approach within the CAR immune cell therapy platform, and adoptive therapy remains the mainstream method for CAR-T treatment: autologous immune cells are extracted from the patient, expanded ex vivo, engineered with CAR molecules, and reinfused into the patient to kill target cells through immunocyte-specific cytotoxic mechanisms, thereby achieving disease remission or improvement [7]. The principal merits of this adoptive therapeutic approach are that it provides a personalized treatment plan to halt disease progression based on the patient’s own immune system, with the potential for the development of “universal” and “off-the-shelf” CAR immunotherapy products [8, 9]. However, this highly individualized approach through the isolation of autologous cells is accompanied by high costs and complex technical barriers, which limit widespread adoption and equal access to CAR immune-cell therapy-both in terms of the rapid production of CAR immune cells and patients’ financial resources [10, 11]. Additionally, prolonged ex vivo culture and manufacturing processes may lead to phenotypic and functional alterations in CAR-T cells, rendering cell products with heterogeneous components and increasing therapeutic risks [12, 13].

**Fig. 1:**
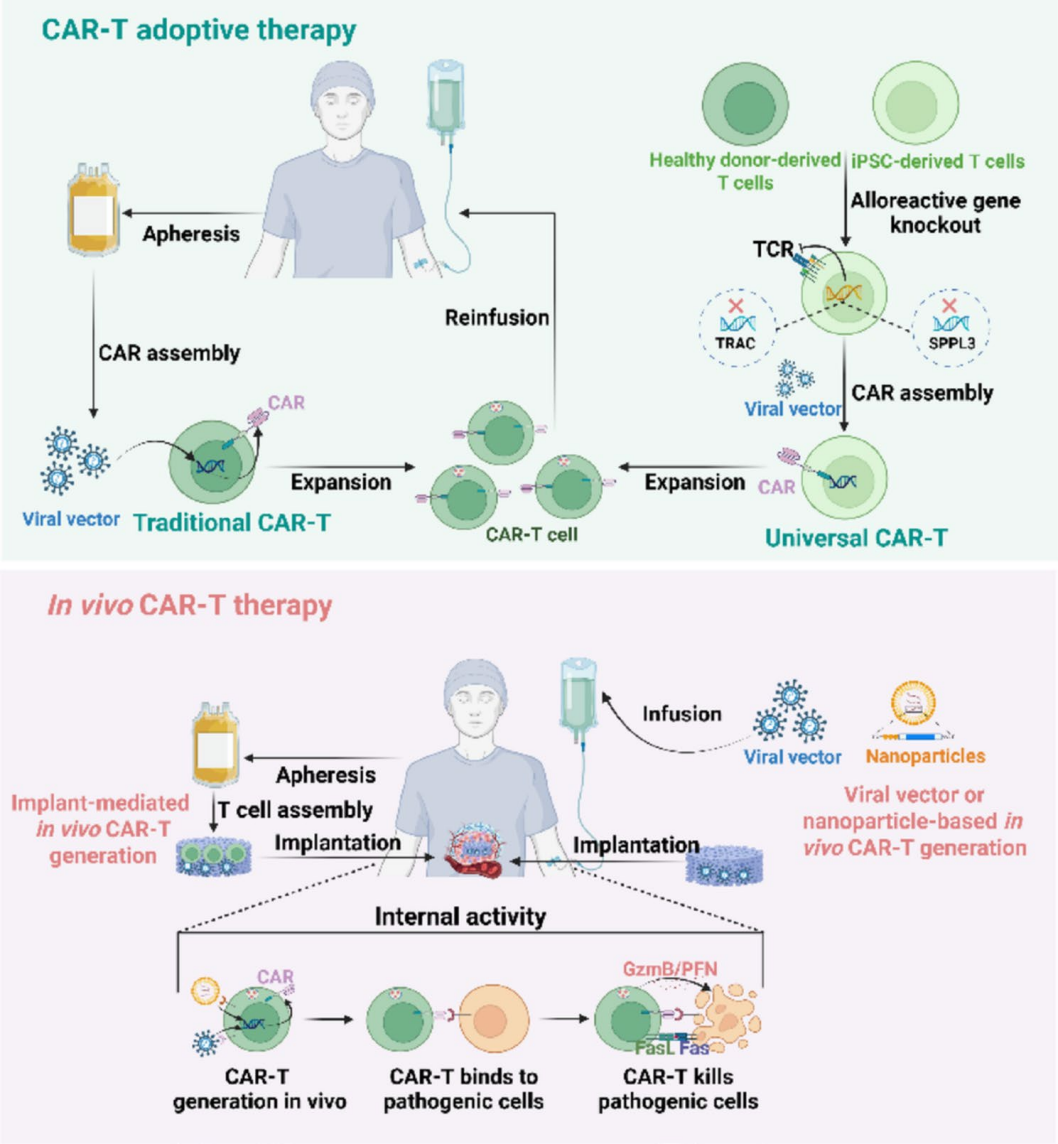
The basic technical pathways of adoptive therapy and in vivo CAR-T therapy. Traditional CAR-T therapy entails harvesting a patient’s autologous T cells, which are then transduced with a CAR gene, expanded ex vivo, and reinfused into the patient to mediate therapeutic effects. In contrast, universal CAR-T therapy represents an advancement in adoptive cell therapy. This approach utilizes T cells derived from healthy donors or induced pluripotent stem cells (iPSCs) that are genetically edited to knock out key genes mediating alloreactivity. These engineered cells subsequently undergo CAR integration and expansion in vitro to generate standardized, “off-the-shelf” therapeutic products. A more emerging paradigm, in vivo CAR-T therapy, bypasses ex vivo manufacturing by directly engineering T cells within the patient’s body. This is achieved through the administration of viral vectors or nanoparticles encoding the CAR gene. Furthermore, innovative strategies employing implantable scaffolds pre-loaded with CAR delivery vectors present an alternative method for the in situ generation of CAR-T cells

In vivo CAR immune cell programming is an interesting idea: a virus or other vector can be used to load CAR genes, target immune cells in vivo, and create CAR immune cells at the site of disease or in the human circulation. This method greatly reduces the production costs and manufacturing timelines of CAR immune cells, avoids the potential therapeutic risks associated with in vitro immune cell production, and represents a promising alternative approach for CAR-T manufacturing [14] (Fig. 1). In addition, although in vivo CAR-T follows different technological paths, this represents only a difference in manufacturing approaches, and the ultimate mode of action is that the CAR-T cells recognize and eliminate target cells with a specific antigen [15]. Therefore, some enhancement strategies and concepts in adoptive therapy can be borrowed by in vivo CAR-T, especially some approaches based on gene editing, which can be completed together with in vivo CAR-T generation.

This article introduces the developmental history of CAR-T therapy, summarizes the research advances in in vivo CAR-T cell programming, compares CAR-T cells generated via in vivo and in vitro approaches, discusses the similarities and differences between these two distinct technical paths for cell therapy, and highlights the guiding significance of the adoptive therapy-based augmentation strategy for programming CAR immune cells in vivo and its broad clinical application prospects.

## The development of CAR-T therapy: from adoptive therapy to in vivo manufacturing

**Fig. 2:**
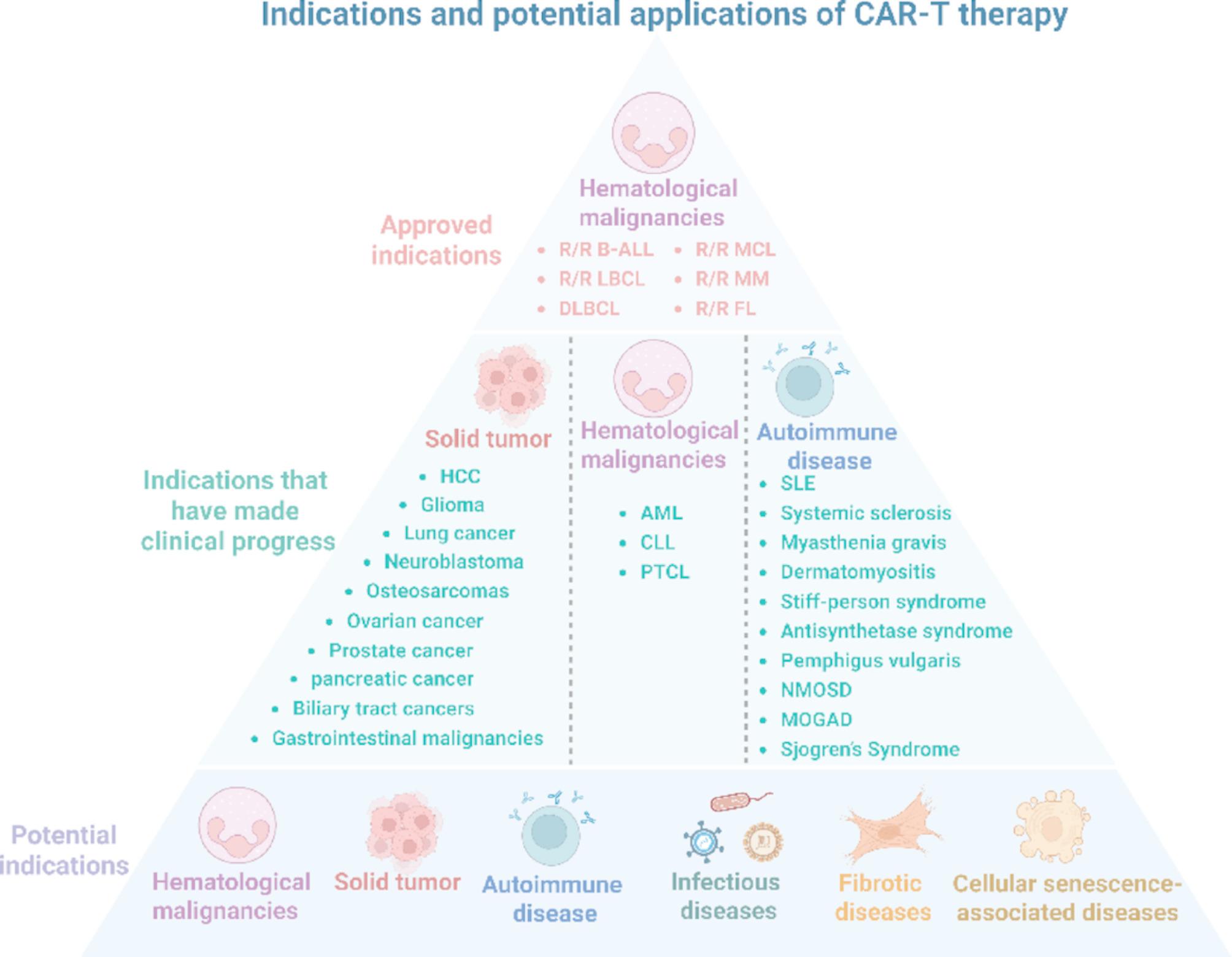
Major modules of CAR molecules and key parameters or features affecting their functions. The CAR molecule mainly consists of four modules, the antigen-binding domain, the hinge region, the transmembrane domain and the intracellular domain, which are affected by a variety of structural parameters and characteristics, and finally form the overall effect

The development of CAR-T has made researchers realize that CAR molecules are a suitable immune cell activation tool, which can break through the conventional MHC-dependent pathway and achieve immune killing by CAR molecular-mediated signal activation [16]. During the development of CAR immune cell therapy, the functions and modifications of each module within the CAR molecule have consistently been a key focus area for researchers. CAR contains four basic structures: an antigen-binding domain, a hinge domain, a transmembrane domain, and an intracellular domain, each of which has unique functions [17]. Since CAR-T was identified as a cell therapy method, the molecular structure of CAR has undergone five generations of changes, and its basic function has also been deeply studied and elaborated (Fig. 2).

**Fig. 3:**
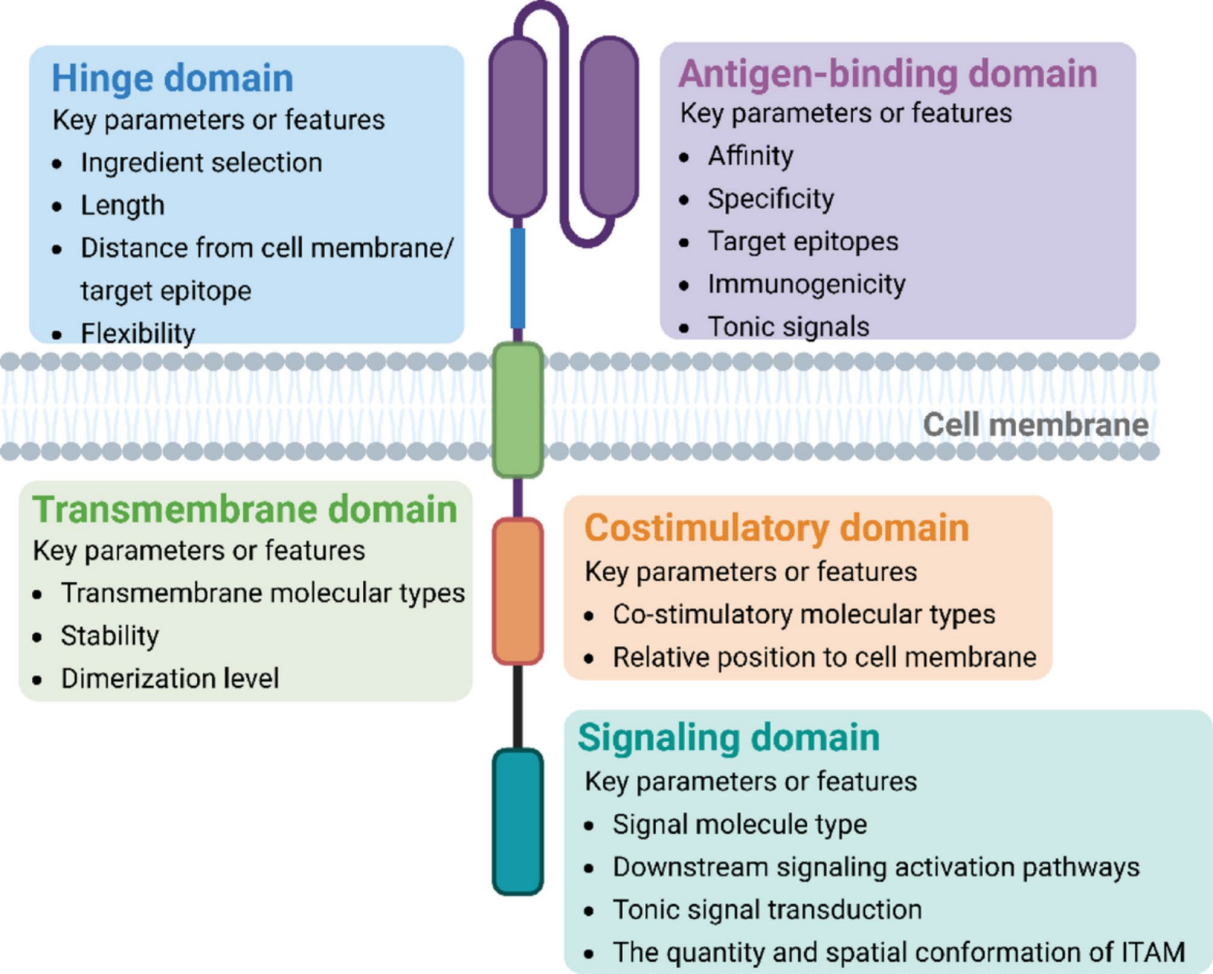
CAR-T therapy has been approved and potential indications. There are six approved indications for CAR-T therapy, focusing on hematological malignancies. In addition, clinical results of clear advances in the treatment of solid tumors, autoimmune diseases and other treatment fields have been reported. The purpose of the triangular structure is to emphasize the expanding indications for CAR-T therapy and its broad applicability: potential applications for related diseases that achieve remission or cure by eliminating pathological cells. *B-ALL* B-cell acute lymphoblastic leukemia, *MCL* Mantle cell lymphoma, *LBCL* Large B-cell lymphoma, *MM* Multiple myeloma, *DLBCL* Diffuse large B-cell lymphoma, *FL* Follicular lymphoma, *HCC* Hepatocellular carcinoma, *AML* Acute myeloid leukemia, *CLL* Chronic lymphocytic leukemia, *PTCL* Peripheral T-cell lymphoma, *SLE* Systemic lupus erythematosus, *NMOSD* Neuromyelitis optica spectrum disorder, *MOGAD* MOG-IgG associated disorders, *R/R* Relapsed/Refractory

CAR-T therapy currently represents the most advanced therapeutic modality among CAR-based immunotherapies, with a developmental history spanning over 35 years, this approach achieved a breakthrough in clinical hematologic malignancy treatment in 2011, subsequently propelling substantial attention toward its therapeutic product development [18]. To date, multiple therapeutic products have been approved and commercialized, offering curative potential for patients with leukemia and lymphoma. At the same time, CAR-T cell therapies have shown remarkable clinical potential across a spectrum of refractory or therapeutically underserved disease domains beyond oncology (Fig. 3) [19, 20], and the therapeutic form has also experienced a tortuous process from adoptive therapy of autologous cells and allogeneic cells to in vivo manufacturing [21].

Beyond oncology, CAR-T therapy has shown clinical-grade application potential in the treatment of a variety of diseases. However, the number of patients who can receive CAR-T therapy is still a minority, which is mainly limited by the complex manufacturing process of CAR-T, poor quality of patients’ autologous immune cells, and the unacceptably high cost [22]. In addition, unlike conventional drugs, CAR cell therapy is a highly personalized therapy, and the in vivo process after infusion is entirely “spontaneous”, which represents unpredictable pharmacokinetics, potential risk of adverse events, and uncertainty in therapeutic efficacy [23].

Therefore, it is urgent to develop a CAR immune cell therapy strategy that patients can afford and is easy to standardize production. In related studies, universal CAR-T therapy and in vivo CAR-T manufacturing are currently the two mainstream methods, which represent two different technical paths. The universal CAR-T still follows the basic principles of adoptive therapy, knocking out key genes of alloresponse by gene editing [24]. This approach expands the cell source for adoptive therapy and solves the problem of inadequate quantity and poor quality of T cells in patients, while providing elements for standardized production so that CAR-T therapy is no longer limited by production time. However, the cost of universal CAR-T is still high, in vitro production still carries therapeutic risks, and in vivo processes are made more unpredictable by the influence of allogeneic cell sources [25]. In addition, immune rejection issues such as graft-versus-host disease (GVHD) and host-versus-graft reaction have not been fully addressed. Although immune rejection can be controlled to a certain extent by knocking out the TCR receptors on the surface of αβT cells or using non-αβ T cells or virus-specific memory T cells, these strategies often lead to new problems and are difficult to achieve clinical application [26].

Beyond allogeneic CAR-T cells derived directly from donors, induced pluripotent stem cells (iPSCs) have emerged as a potential alternative for “off-the-shelf” CAR-T therapy due to their unlimited proliferation capacity, ability to differentiate into various adult cell types, ease of genetic editing, relatively mature operational protocols, potential for large-scale production, and freedom from ethical constraints [27]. Although iPSC-derived CAR-T cells still carry a risk of immune rejection, the development of rejection-free universal iPSC CAR-T cells has been achieved through gene knockout strategies [28–30]. Multiple engineering modifications can further enhance their potent antitumor effects within the tumor microenvironment [31, 32]. However, several challenges require in-depth investigation, including ensuring the stable large-scale expansion and directed differentiation of iPSCs in vitro, guaranteeing that the generated T cells possess antitumor potential comparable to that of natural T cells, and addressing the tumorigenic risk associated with residual undifferentiated iPSCs [33]. Additionally, strategies such as reducing ex vivo culture time and shifting toward decentralized or hospital-based production have been proposed. However, their implementation still faces challenges due to complex ex vivo manufacturing processes and high costs [21, 34]. In summary, for CAR-T adoptive therapy, researchers have been working hard to shorten the production time, simplify the preparation process and reduce the related costs [35].

In these contexts, and leveraging advances and convergence in multidisciplinary fields such as gene delivery and editing technologies, RNA-based drug development, and CAR immune cell therapy, the concept of in vivo CAR-T manufacturing has been proposed and extensively investigated. There is no essential difference in the therapeutic mechanism between in vivo CAR-T and adoptive CAR-T, but the in vivo manufacturing approach addresses most of the drawbacks of adoptive therapy, including poor patient accessibility, complex manufacturing process, high cost, challenges in providing “standardized” therapeutic products, and difficulties in transportation [36] (Table 1). Remarkably, relevant clinical studies have advanced, and key CAR gene delivery technologies have also made progress. In addition, the indications of CAR-T in vivo are not limited to cancer, refractory diseases such as autoimmune diseases and fibrosis are also advancing simultaneously [15, 37].

**Table 1:** Comparison of different types of CAR-T cells

Dimension	Traditional CAR-T	Universal CAR-T	In vivo CAR-T
Cell source	Isolation of autologous T cells and in vitro expansion	PBMCs from healthy donors; iPSC	Editing in vivo in patients
Preparation time	3–6 weeks to complete product preparation and re-infusion	Preparation in advance and immediate infusion	Preparation in advance, immediate administration, 10–17 days to reach the peak amplification
Relative cost	High (may decrease in the future)	Moderate	Low
Clinical toxicity manifestations	CRS; ICANS; hematotoxicity; potential long-term side effects (Secondary infection; SPMs)	GvHD; CRS; ICANS; hematotoxicity; Secondary infection	CRS; ICANS; hematotoxicity; Secondary infection
Persistence	Intermediate to long (months to years)	Short to intermediate (weeks to months)	Unknown (insufficient data)
Phenotypic control ability	High, specific phenotypes can be induced by in vitro preconditioning	High, specific phenotypes can be induced by in vitro preconditioning	Low, limited ability to control phenotype in vivo
Technology maturity	High, multiple products have already received market approval	Low, still in clinical studies	Low, still in clinical studies

*CAR* chimeric antigen receptor, *CRS* cytokine release syndrome, *GVHD* graft versus host disease, *ICANS* immune effector cell-associated neurotoxicity syndrome, *iPSC* induced pluripotent stem cel, *SPMs* secondary primary malignancies

## Manufacturing methods for in vivo CAR-T cells

The development of in vivo CAR-T therapy is based on advances in gene delivery and editing technologies. Unlike adoptive therapy, the targeting specificity, delivery efficiency, and editing accuracy of CAR gene delivery determine therapeutic efficacy and safety of this therapeutic approach during the generation of in vivo CAR-T cells. Therefore, the selection of appropriate delivery vectors and methods is the key step in the manufacture of CAR-T cells in vivo. Currently, three distinct technical platforms have been employed for in vivo CAR-T cell generation, each exhibiting unique characteristics (Table 2).

**Table 2:** Comparison of three platforms of CAR-T cell generation

Dimension	Virus-based vector platforms	Nanoparticle-based carrier platforms	Implant platform
Platform carrier type	Lentivirus; Retrovirus; Adeno-associated virus	LNPs with different formulations	Implants containing CAR virus vectors
Immunogenicity	High, limit repeat dosing	Low, supporting repeat dosing	Low, use base material with high biocompatibility
Potential risk	Off-target delivery; Non-targeted toxicity; Genomic integration error	Off-target delivery; Non-targeted toxicity; Dose-dependent toxicity	Non-targeted toxicity; Genomic integration error
Delivery efficiency	Targeted clear, but limited packaging capacity	Antibody modification enables precise targeting and packaging of large mRNA fragments	In-scaffolds delivery
In-vivo editing efficiency	Continuous expression, but may increase the risk of off-target	Ransient expression, persistence may be limited	In-scaffolds editing with high efficiency
Technical progress	Preliminary clinical trial results were obtained	Clinical safety testing based on healthy volunteers	Pre-clinical stage

*CAR* chimeric antigen receptor, *LNPs* lipid nanoparticles

### Viral vector-based in vivo CAR-T generation

In adoptive therapy, CAR gene delivery methods have been well developed, mainly including viral-based and nanomaterial-based delivery approaches. Lentiviral vectors are the most widely used and first clinically approved CAR gene delivery system for ex vivo CAR-T production, which can stably integrate the CAR gene into the host cell genome [38]. However, for in vivo CAR-T manufacturing, this approach faces substantial uncertainties, including host immune responses against lentiviruses, lack of targeting to immune cells, and insertional mutagenesis risks [39].

Nevertheless, considering its excellent performance in adoptive therapy, researchers are still working hard to achieve lentivirus-based in vivo CAR-T manufacturing. VivoVec is a lentiviral vector-based platform designed for the in vivo generation of CAR-T cells, with multiple candidate delivery vectors under development. UB-VV100, a clinical candidate from this platform for treating B-cell malignancies, incorporates a CAR gene delivered via lentivirus and features a rapamycin-activated cytokine receptor (RACR) system. This RACR system converts rapamycin binding into IL-2/IL-15 signaling, thereby promoting the proliferation of the in vivo CAR-T cells. In pre-clinical studies, UB-VV100 demonstrated dose-dependent activation and transduction, enabling the stable generation of CAR-T cells in vivo without the need for prior lymphodepletion. These cells effectively targeted and eliminated tumor cells, while also exhibiting favorable biodistribution and safety profiles [40].

Furthermore, the platform employs an innovative strategy to enhance in vivo transduction and function by displaying a multi-domain fusion (MDF) protein on the surface of the lentiviral vector. This engineered MDF protein combines an anti-CD3 scFv for T-cell activation with the costimulatory molecules CD80 and CD58. Consequently, upon binding to a T-cell, the vector simultaneously provides both primary activation and costimulatory signals, leading to more efficient in vivo CAR-T cell generation. In non-human primate models, these MDF-modified VivoVec particles mediated robust in vivo CAR-T cell generation, resulting in sustained B-cell depletion. Notably, following B-cell repopulation, CAR-T cells in the peripheral blood rapidly expanded and re-eliminated B-cells, demonstrating the establishment of long-term immunological memory [41]. This study presents a novel therapeutic strategy for B-cell malignancies and autoimmune diseases, advancing the field of in vivo CAR-T cell therapy and introducing a new methodology for the surface modification of lentiviral vectors.

The precise delineation of T-cell subsets relies on specific combinations of surface molecules. It has been established that constructing CAR-T products by targeting T-cell subsets with specific surface markers, such as CD62L, CCR7, can significantly enhance their persistence and anti-tumor efficacy [42, 43]. Pfeiffer et al. developed a CD8-targeting lentiviral vector that successfully generated CD8^+^ CAR-T cells in mice, and effectively eliminated lymphoma cells [44]. After that, the research team proposed the idea of constructing CD4^+^ CAR-T cells in vivo and realizing it by CD4-targeting lentivirus. CD4^+^ CAR-T cells exhibited superior in vivo activity compared to CD8^+^ CAR-T cells, possibly because CD8^+^ T-cells were more easily exhaustion [45]. These two studies provide novel lentiviral-based approaches for in vivo CAR-T cell generation and highlight the critical importance and selectivity of T-cell subtypes during in vivo CAR-T manufacturing. However, exerting precise control over the phenotypic fate of CAR-T cells manufactured in vivo remains challenging due to the limited intervenability of the manufacturing process. Therefore, a promising strategy to potentiate CAR-T cell function involves targeting these specific markers to generate products enriched with less-differentiated phenotypes in vivo.

Furthermore, the development of novel universal lentiviral delivery platforms has provided a versatile approach to advance this concept. James I. Andorko and colleagues designed a fusion protein that enables retargeting of lentiviral vectors. By introducing site-specific amino acid mutations, they ablated the binding capacity of VSV-G to its native receptor, LDL-R, without compromising its fusogenic activity. Building on this, the incorporation of specific binders (such as CD3, CD4, or CD7) enabled highly efficient in vivo transduction of target cell types, including T-cells and NK cells [46]. This strategy not only improves vector safety but also provides a robust tool for in vivo engineering of CAR immune cells. Such precise viral engineering approaches enhance control over the in vivo generation of CAR-T cells, thereby opening new avenues for personalized immunotherapy.

In addition, adeno-associated viruses (AAV) have been used for in vivo CAR-T production due to their superior performance in therapeutic gene delivery. The use of AAV in vivo CAR-T therapy is supported by the study of Nawaz et al., who used a modified AAV vector to successfully reprogram immune cells in mice to express CAR molecules in vivo, resulting in effective tumor regression and the development of anti-tumor immune characteristics [47].

It is worth emphasizing that the risk of insertion mutagenesis must be paid attention to for viral vectors, in current clinical practice, there has been a case of related factors leading to patient death: a patient who received adoptive therapy with anti-BCMA CAR-T cells developed secondary gastrointestinal T-cell lymphoma. This occurred because the CAR gene integrated into the T-cell genome at a site within the TP53 gene, disrupting its tumor-suppressing function, ultimately leading to malignant transformation and uncontrolled proliferation of the CAR-T cells [48]. This risk requires careful consideration in viral vector-based in vivo CAR-T studies, and the corresponding identification and detection should be carried out before treatment, but it is foreseeable that this will increase the already high related costs.

### Nanoparticle-based in vivo CAR-T generation

Lipid nanoparticles (LNPs) and other nanoparticles can effectively deliver nucleic acids to target cells in vivo while avoiding degradation and clearance. Compared with viruses, nanoparticles are easier to be engineered to target T-cells, have high safety, and are relatively cheaper. Importantly, unlike viral vectors, nanoparticle-delivered mRNA undergoes cytoplasmic translation without insertional mutagenesis risks [49]. In addition, nanoparticles have potential industrial production value for easy large-scale production, transportation and storage.

Currently, LNPs with various formulations represent the primary nanomaterial platform for in vivo CAR-T cell generation. Conventional LNPs are typically composed of four constituent classes: an ionizable lipid, a phospholipid, cholesterol, and a PEG-lipid [50]. For in vivo CAR-T cell engineering, the LNP formulation is often optimized. This includes strategies such as conjugating targeting moieties to the LNP surface to confer T cell-specific tropism and to modulate surface properties.

As early as 2017, Smith et al. proposed the idea of using synthetic DNA nanocarriers for in situ editing of T-cells. They achieved T-cell targeting by incorporating anti-CD3 fragments on the surface of biodegradable poly (β-amino ester)-based nanoparticles, CAR gene integration was achieved via a transposon system, and the experimental results support the feasibility of in situ CAR-T programming to combat cancer, although the overall editing efficiency remains relatively low [51]. The development of LNP-mRNA delivery technology based on COVID-19 treatment has brought new opportunities for immune cell therapy. Rurik et al. explored the potential of this technology for in vivo CAR-T manufacturing, CD5-targeted LNP successfully delivered CAR-mRNA into murine T-cells and expressed the CAR molecule. This approach achieved targeted clearance of fibroblasts, improved cardiac function in model mice, and expanded the potential indications for in vivo CAR-T therapy [52]. Similarly, Li et al. constructed CAR-LNP containing mRNA encoding both a CAR and IL-7, successfully edited CAR-T cells expressing CAR and IL-7 in mice and eliminated melanoma cells in vivo, suggesting that the enhanced strategy involving cytokine co-expression can also be applied to in vivo CAR-T therapy [53]. The latest targeted lipid nanoparticle (tLNP) technology developed by Haig Aghajanian’s team enables in vivo editing of CAR-T cells while reducing off-target delivery to the liver [54]. This approach demonstrated favorable safety and tolerability profiles across multiple animal models. In a humanized leukemia mouse model, tLNP treatment significantly suppressed tumor growth, with some animals achieving complete tumor eradication. Surprisingly, in cynomolgus monkeys, this in vivo CAR-T technology induced rapid and profound B-cell depletion, followed by recovery after treatment cessation. This breakthrough may offer new hope for treating autoimmune diseases by achieving long-term disease remission through transient depletion of pathogenic B-cells [55].

Due to the limitations of mRNA in delivery efficiency and in vivo persistence, circRNA and DNA have been explored as alternative platforms for in vivo CAR-T cell generation. Like mRNA, circRNA does not require genomic integration; however, its circular conformation confers greater stability than linear mRNA, enabling prolonged protein expression [56]. Wang et al. [57] developed an LNP-mediated circRNA delivery system for in vivo immune cell engineering. By using LNPs to deliver circular RNAs encoding CAR molecules, their approach generated a broad population of CAR-expressing cells in vivo, sustained CAR expression for over 72 h, effectively suppressed tumor growth in animal models, and remodeled the tumor microenvironment. Beyond RNA, DNA-based systems have also been developed for in vivo programming of CAR-T cells. Bimbo et al. [58] established an LNP platform for in vivo CAR-T cell production via DNA delivery. This system co-delivers minicircle DNA encoding the CAR construct and transposase mRNA using a single targeted nanoparticle, facilitating stable genomic integration and durable CAR expression in T-cells.

LNP-mediated targeted delivery typically relies on antibody modification; however, this approach faces challenges related to antibody selection, modification density, and conjugation efficiency [59]. Poor antibody choice may accelerate LNPs clearance or cause overactivation of target cells [59]. In this context, naturally T cell-tropic lipid formulations, such as those based on cardiolipin—have emerged as promising alternatives. Zhang et al. [60] reported a novel strategy using antibody-free, cardiolipin-mimetic lipid nanoparticles for in vivo CAR-T cell engineering. By incorporating a cardiolipin-mimetic phosphoamine lipid into the LNP formulation, they enhanced T-cell-targeted delivery in vivo through increased particle stiffness and phase separation. Moreover, the use of circular RNA further extended CAR molecule expression. In models of liver fibrosis and rheumatoid arthritis, in vivo-generated CAR-T cells edited by this platform successfully eliminated senescent fibroblasts, providing proof-of-concept for the application of in vivo CAR-T cell therapy in inflammaging-related diseases.

In summary, nanoparticle-based platforms for in vivo CAR editing continue to evolve in response to clinical demands. However, challenges remain in areas such as persistence in vivo, immunogenicity, and editing efficiency. Furthermore, the pharmacokinetic properties of in vivo-generated CAR-T cells require further elucidation through additional clinical data.

### Implant-mediated CAR-T generation in vivo

In addition to in vivo immune cell-targeted delivery by direct injection of virus, nanoparticles, or LNP, the implantation of scaffolds to establish a localized in vivo delivery platform represents an interesting alternative approach. Currently, this implant-based CAR-T generation approach comes in two main forms. The first is to isolate T-cells from the patient’s blood in advance, encapsulate them in a scaffold with a CAR gene delivery vector, and then implant them into the tumor to complete the production of CAR-T in vivo. Although this method has the process of isolating T-cells in vitro, this phase is relatively brief, and the activation and expansion of T-cells are not carried out in vitro, CAR-T cells are ultimately produced in vivo, so it is also considered as a kind of in vivo CAR-T manufacturing technology. For example, Yevgeny Brudno’s team developed an implantable, multifunctional alginate scaffold for T-cell engineering and release. This scaffold incorporates retroviral particles; when patient-isolated T-cells enter it, they are engineered to express CAR molecules and stimulated to proliferate and activate by cytokines contained within the scaffold. Ultimately, the scaffold releases sufficient quantities of fully functional CAR-T cells into the lesion site to perform their therapeutic function. Notably, in tumor rechallenge models, scaffold-generated CAR-T cells showed superior efficacy compared to intravenously administered CAR-T cells due to their better in vivo expansion and persistence [61]. Further, the team developed an alginate-based porous biomaterial scaffold (Drydux) that enables rapid and efficient in vivo generation of tumor-specific CAR-T cells in just 3 days from patient blood collection to completion of CAR-T production, with long-lasting in vivo release. Drydux-generated CAR-T cells were significantly more effective than adoptive CAR-T cells in animal models of systemic lymphoma, peritoneal metastatic ovarian cancer, intravascular metastatic lung cancer, and orthotopic pancreatic cancer, producing durable tumor responses [62].

Another approach is to implant implants containing a CAR gene-delivery vector into the disease site to generate CAR-T cells by recruiting T-cells in vivo into the inside of the scaffold for gene editing and expansion. Inamdar et al. demonstrated another possibility for implant-based CAR-T production in vivo by using the biomaterial implant approach: the implant recruited immune cells, and lentiviral vectors contained in porous collagen matrix assembled CAR molecules to T-cells, which were subsequently rapidly expanded and released into the surrounding tumor tissue. In animal models, this approach significantly inhibited tumor development and prolonged the survival of mice [63]. Zhu et al. developed an injectable supramolecular hydrogel system that enables in situ generation and efficient accumulation of CAR-T cells when injected around solid tumors. In addition, this platform also induces a strong inflammatory response and improves the immunosuppressive microenvironment of solid tumors, with strong anti-tumor efficacy and reliable safety profile [64]. Compared with direct injection of CAR gene vectors, this way of recruiting immune cells through biomaterials limits the contact between CAR gene vectors and T-cells, and controls its expansion, which can produce enough CAR-T cells in the disease area in a short time. However, this treatment modality may not be suitable for patients with diffuse lesions or hematologic disorders and is limited by the quality of the patient’s immune cells.

In summary, these implant-based in vivo CAR-T cell generation approaches offer a novel strategy: leveraging biomaterials to create an in situ platform where T-cells undergo CAR molecule integration, expansion, and activation. This partially overcomes limitations in CAR-T cell quantity while reducing the risk of off-target CAR expression in non-T cells. In addition, compared with adoptive therapy or systemic vector distribution methods, this in vivo platform has the ability to continuously program and release CAR-T cells, which can effectively achieve the quantitative accumulation of CAR-T cells in the local disease, with better therapeutic efficacy and safety. It is hopeful to be developed as a universal CAR immune cell engineering method.

## Advantages and characteristics of in vivo CAR-T manufacturing

### Streamlining manufacturing and therapeutic processes

**Fig. 4:**
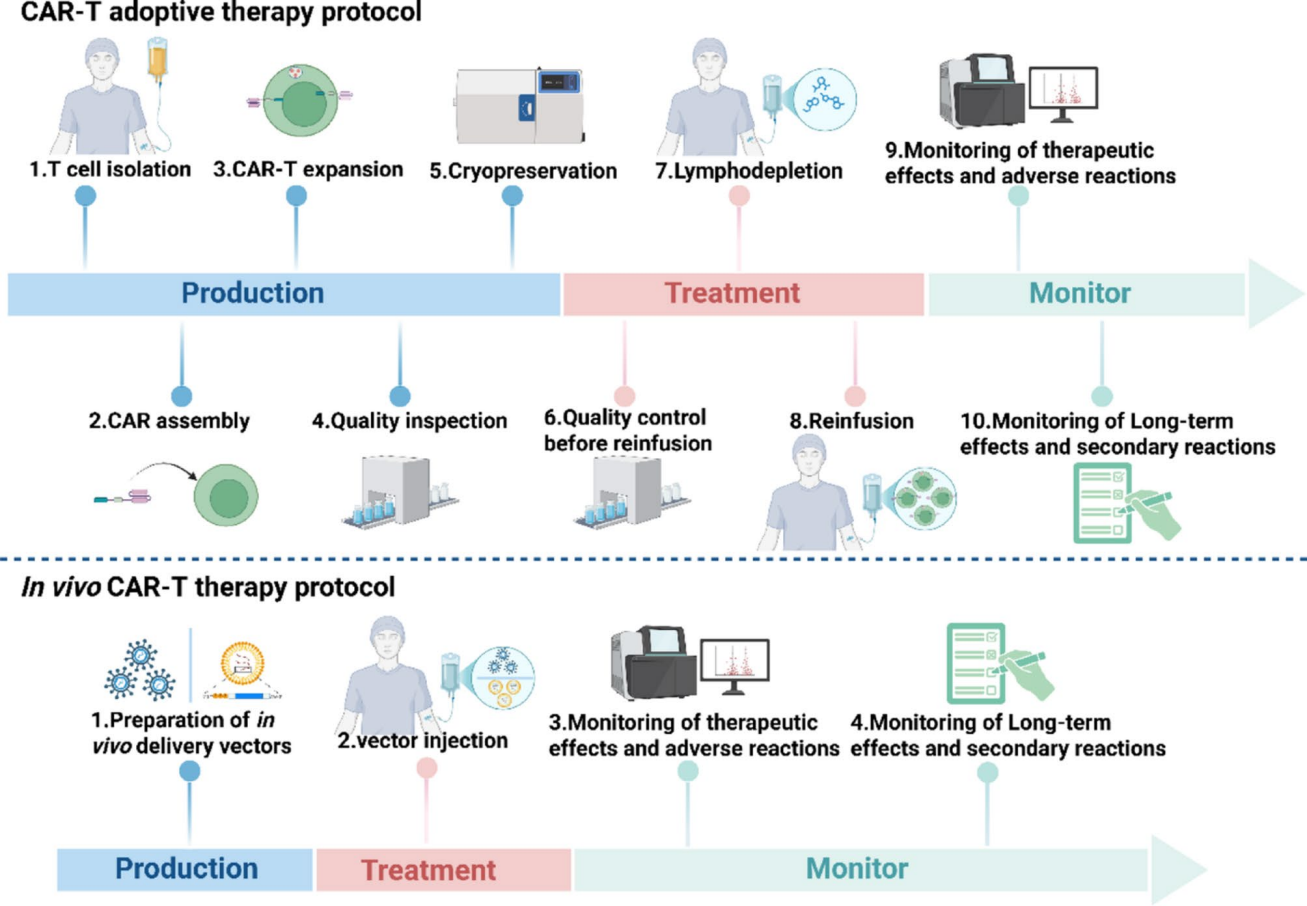
This figure provides the management process for adoptive therapy and in vivo CAR-T therapy. In addition to streamlining the treatment process, facilitating rapid delivery of CAR-T therapeutic products and enhancing patient accessibility are the most attractive benefits of in vivo CAR-T

A significant advantage of in vivo CAR-T therapy is that it its potential to serve as a universal treatment, CAR vector drugs with clear physical and chemical properties can be used for a large number of patients with CAR-T treatment needs [65]; at the same time, compared with adoptive cell therapy products, CAR vector drugs offer significant advantages in manufacturing, storage, transportation, and quality control, which is conducive to promoting the large-scale implementation of CAR immune cell therapy [21]. In terms of treatment process, in vivo CAR-T greatly simplifies the complex procedures of adoptive therapy (Fig. 4), avoids the lymphodepletion chemotherapy and repeated quality check of CAR-T products, and reducing the time from treatment decision to administration. This high efficiency is particularly critical for rapidly progressing acute malignancies or patients with advanced-stage disease [62]. Furthermore, in vivo CAR-T therapy has been shown to induce long-term immune memory, which may enable more precise restoration of immune homeostasis in patients with autoimmune diseases [41].

### Reduce costs and improve patient access

In the current adoptive therapy, CAR-T therapy generally takes about 10–25 days from cell collection to infusion, and the whole process depends on manual operation, which is difficult to scale up [22]. In addition to operational methods, materials and production environment, the quality of patient’s autologous immune cells is the most difficult variable to control in CAR-T manufacturing [66]. Cell viability, T-cell number and phenotype, and health status profoundly affect the treatment outcome and are related to the success rate of product manufacturing [66]. An exploratory study showed that the cost of CAR-T therapy for patients who received CAR-T therapy and had a positive outcome was around $110,000–120,000 [67]. Similarly, the study by Jagannath et al. provided data on the cost of anti-BCMA CAR-T therapy in patients with multiple myeloma, the average cost per patient over 12 months was $160,933 (excluding CAR-T cell acquisition costs) [68]. Obviously, under the current limitations of personalized production and complex process conditions, it is difficult for most patients eligible for CAR-T treatment to obtain treatment opportunities without high cure rate and heavy economic burden [69].

Compared to adoptive cell therapy, in vivo CAR-T therapy is anticipated to yield substantially lower manufacturing costs. This reduction stems from a simplified production process that eliminates complex ex vivo expansion, reduces the need for preconditioning (such as lymphodepletion), and shifts from patient-specific regimens to “off-the-shelf” products, thereby significantly decreasing the cost per dose. However, these advantages may be counterbalanced by considerably higher research and development expenses. Overcoming key challenges, such as off-target effects, host immune rejection, and limited transduction efficiency, will require substantial investment. The ultimate cost-effectiveness of this approach will necessitate validation through robust clinical data. However, the simplified production and treatment process of in vivo CAR-T therapy provides an opportunity for large-scale production, presents a scalable alternative that could substantially reduce costs and improve accessibility.

### Efficacy and safety advantages

A key procedural difference between in vivo CAR-T and adoptive therapy involves lies in the administration of lymphodepleting preconditioning. Lymphodepleting preconditioning involves cytotoxic chemotherapy/radiotherapy to eliminate host lymphocytes before adoptive CAR-T infusion [70]. The aim is to promote the adaptation of CAR-T cells and eliminate immunosuppressive cell types, such as regulatory T-cells and myeloid-derived suppressor cells, for maximum therapeutic benefit [70]. However, this procedure carries risks including infection, leukopenia, and pulmonary veno-occlusive disease. Furthermore, treatment-induced reactive myelopoiesis may amplify inflammatory cytokine responses, driving excessive IL-6 production and CRS initiation [71, 72]. Due to the dependence of in vivo-generated CAR-T therapy on endogenous T-cell production, this therapeutic approach necessitates preservation of the patient’s lymphocytes. Therefore, in vivo CAR-T that avoids lymphodepleting preconditioning is superior to adoptive therapy in terms of safety, and preserving the intact immune system may better support epitope diffusion and the generation of a broad anti-tumor immune response, thereby preventing antigen escape [73]. While potentially reducing efficacy (such as immune-mediated CAR-T clearance), ex vivo expanded CAR-T cells exhibit greater exhaustion risks and risk of heterogeneity, whereas in vivo CAR-T cells may have advantages in stemness and proliferation, yielding superior in vivo activity at lower doses [74].

## Challenges associated with achieving in vivo CAR-T therapy

**Fig. 5:**
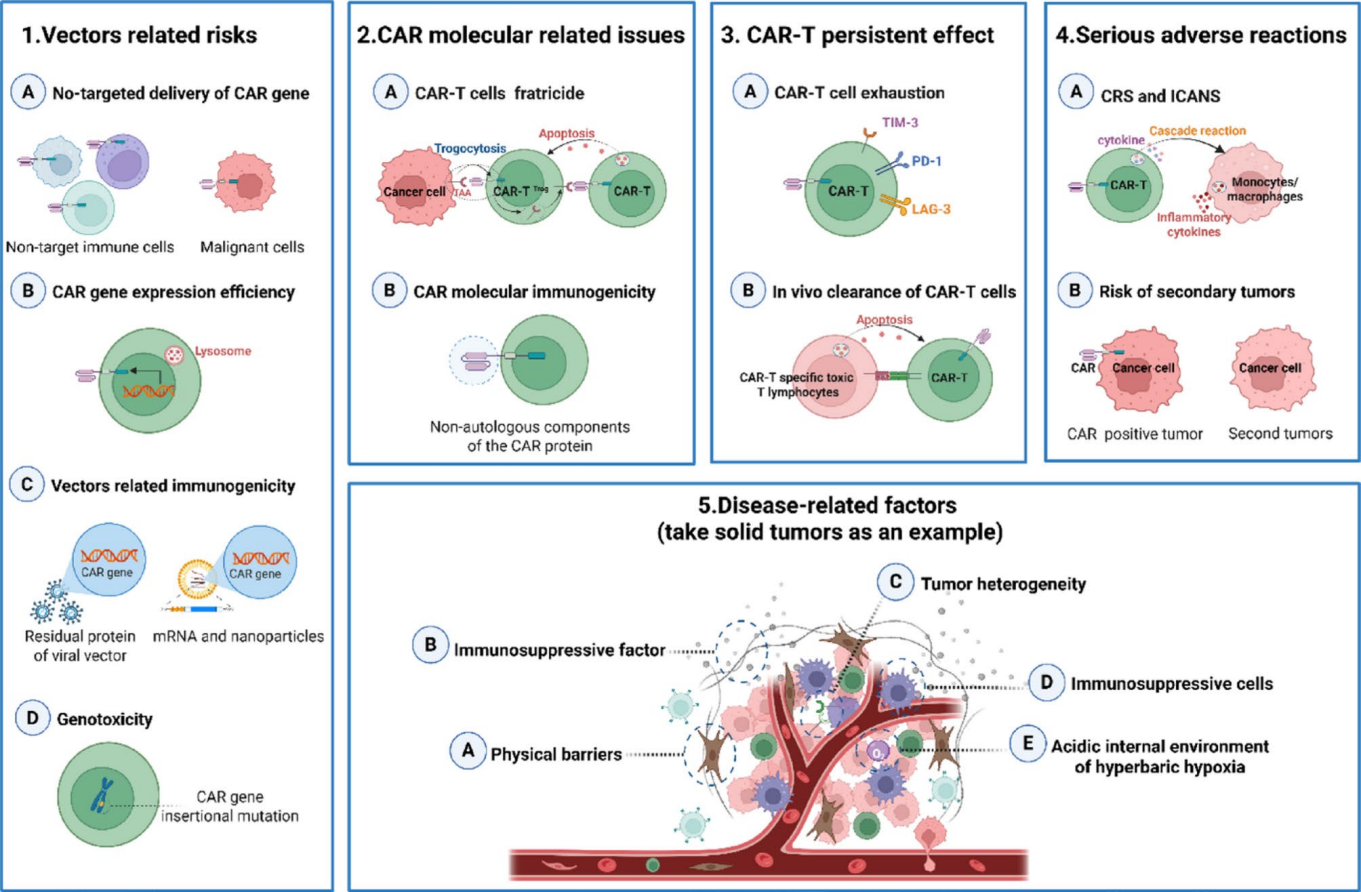
Potential risks and related barriers to the realization of in vivo CAR-T therapy. The main sources of these limitations include the in vivo delivery vector, the intrinsic properties of the CAR molecule and T-cells, and disease-related factors

For in vivo or in situ CAR-T programming to be of therapeutic significance, several prerequisites must be met: (1) The CAR gene must be accurately delivered into and expressed within the target immune cells; (2) The limited number of CAR immune cells generated in vivo must possess robust effector functions or expansion capabilities; (3) The adverse effects caused by an uncertain number of CAR immune cells must be acceptable. Although in vivo CAR-T therapy has demonstrated efficacy in pre-clinical models (e.g., oncology and fibrosis) and holds translational promise, its development as a novel immunotherapeutic modality faces several challenges, including technical, biological, and clinical dimensions (Fig. 5). Furthermore, to date, few in vivo CAR-T trials have reported safety outcomes; thus, these concerns remain theoretical and require prospective monitoring.

### Technical challenges in in vivo CAR-T

#### Potential risks of CAR gene delivery

As mentioned earlier, a critical step in in vivo CAR-T manufacturing is the targeted delivery of the CAR gene to T-cells. However, this process carries the risk of off-target delivery, where the CAR gene is introduced into other cell types. While the consequences of such off-target effects remain poorly characterized, non-specific CAR expression is generally avoided to mitigate uncontrolled risks. Notably, some researcher advocate for CAR-PBMC therapy, hypothesizing that polyclonal CAR immune cells could synergize to enhance anti-tumor responses. However, this approach lacks in vivo validation, with supporting evidence limited to in vitro studies [75]. More alarmingly, the CAR molecule could be erroneously delivered into malignant cells, particularly cancer stem cells. A relevant case was reported in 2018: during CAR-T production, the CAR gene was accidentally introduced into cancerous B-cells and expressed, leading to the proliferation of CAR-positive tumor cells. These cells bound to target antigens on other tumor cells, creating antigen masking that prevented CAR-T cells from recognizing and killing the tumor, ultimately resulting in relapse [76]. Researchers are actively exploring solutions to this issue. For instance, EsoBiotec has developed a T-cell-specific promoter derived from endogenous human gene regulatory elements, enabling functional CAR expression in T-cells. Compared to the EF1α promoter, it demonstrates enhanced T-cell-specific delivery in testing.

#### CAR expression efficiency and regulation

Beyond off-target effects, CAR gene expression efficiency is another critical factor limiting therapeutic efficacy. Viral vectors exhibit high gene transduction efficiency due to their inherent properties, but in clinical applications of adoptive CAR-T therapy, the transfection efficiency of primary T-cells is still difficult to reach the expected level [77]. Nicolai et al. developed a lentiviral platform incorporating CD80 and CD58 costimulatory proteins for in vivo CAR-T generation, which showed enhanced transduction and anti-tumor functionality [41]. The ImmuFron team designed an AI-driven mutant MxV glycoprotein (MxV-G)-pseudotyped lentiviral vector capable of stable T-cell targeting. Compared to traditional VSV-G-pseudotyped vectors, it exhibits higher viral titers and superior transduction efficiency, significantly improving the in vivo generation of CAR-T cells.

In contrast to lentiviral vectors with stable integration capabilities, nanoparticle-based gene delivery is often transient, requiring repeated dosing during treatment. This provides a window for flexible regimen adjustments to maximize clinical benefits [78]. However, their inefficiency, due to mRNA degradation or nuclear delivery requirements for plasmids, limits clinical utility [79].

Recent technological advances show promise for enhancing nanoparticle-based in vivo CAR-T cell manufacturing, efficacy, and controllability. Self-amplifying mRNA (saRNA) technology provides a platform for efficient in vivo generation of CAR-T cells at low doses [80]. Unlike conventional non-amplifying linear mRNA, saRNA encodes a viral replicase that enables intracellular self-amplification, leading to robust and sustained transgene expression from minimal initial input. This characteristic reduces both manufacturing costs and the required payload of delivery vehicles, potentially mitigating dose-related toxicities [80]. However, it should be noted that the double-stranded RNA intermediates produced during saRNA replication resemble viral genetic material and may elicit innate immune activation, raising potential immunogenicity concerns [81]. In parallel, progress in synthetic biology has enabled precise spatiotemporal control over CAR expression. Engineered tunable promoter systems now allow for multidimensional regulation of transgene expression, which is particularly valuable in the context of in vivo CAR-T cell production: such systems facilitate fine-tuning of CAR expression levels to optimize the balance between therapeutic efficacy and safety [82].

### Biological considerations for in vivo CAR-T application

#### Immunogenicity

The immunogenicity of in vivo CAR-T therapy originates from multiple sources, including non-self components of the CAR protein, residual viral vector proteins, mRNA constructs, and their nanocarriers [83]. These factors may induce both humoral and cellular immune responses against CAR-T cells, potentially compromising therapeutic efficacy and creating opportunities for disease relapse. In adoptive CAR-T therapy, immunogenicity-triggered anti-CAR-T responses have been demonstrated to impair the effectiveness of repeat infusions, with expansion of CAR-specific cytotoxic T-cells observed in multiply treated patients [84]. This phenomenon is anticipated to similarly impact in vivo CAR-T therapy outcomes. Notably, since in vivo CAR-T approaches typically omit lymphodepletion protocols, unlike adoptive therapies, they may carry elevated immunogenic risks. While immunogenicity assessment has become standard practice in adoptive CAR-T development, its importance warrants even greater emphasis in in vivo CAR-T platforms [85].

Currently, several strategies specifically aimed at reducing immunogenicity have been proposed. (1) Since the advent of CAR-T therapy, continuous optimization of the CAR structure has been undertaken. Researchers are increasingly using humanized or fully human antibody fragments to replace murine-derived CAR constructs, thereby mitigating immunogenic responses triggered by non-human sequences [86]. (2) The development of viral vectors lacking foreign antigens is critical for in vivo CAR-T generation. Such vectors may reduce immunogenicity, prevent rapid clearance of gene carriers, and allow for repeated dosing [87]. (3) Unmodified mRNA is recognized by intracellular RNA sensors, which can initiate potent innate immune responses, leading to accelerated mRNA degradation and impaired translation [88]. Chemical modification of nucleotides, such as substituting uridine with 5-methoxyuridine, N1-methylpseudouridine, or pseudouridine can diminish immunogenicity and enhance translational efficiency [89]. (4) Optimizing the formulation of LNPs to reduce immunogenicity is also feasible. The development of mRNA vaccines for COVID-19 has provided valuable experience for the in vivo application of LNPs. Relevant studies have found that ionizable lipids in LNPs activate NF-κB and IRF transcription factors through TLR4, thereby regulating innate immune responses. However, by adjusting the lipid composition of LNPs, it is possible to balance the tolerability and stimulatory intensity of mRNA vaccines [90]. This offers new optimization directions for in vivo CAR-T manufacturing based on LNP-mRNA and reducing its immunogenicity.

#### Fratricide

CAR-T cell fratricide presents a clinically significant phenomenon wherein CAR-T cells eliminate each other through recognition of target antigens either natively expressed or acquired on their surfaces [91]. This occurs most prominently in CAR-T therapies targeting T-cell malignancies. Given the scarcity of truly tumor-specific antigens (absent on normal cells) and substantial tumor heterogeneity, pan-T-cell antigens (such as CD5 and CD7) have emerged as the predominant targets in clinical trials for T-cell malignancies [92]. While these targets provide comprehensive coverage against malignant T-cells, they inevitably induce substantial fratricidal killing of both therapeutic CAR-T cells and normal T lymphocytes.

Recent studies have identified a novel fratricide mechanism mediated through trogocytosis, wherein tumor cells transfer membrane antigens (e.g., CD19, BCMA) to CAR-T cells, triggering mutual CAR-T cell attack and subsequent exhaustion [93]. Although this antigen transfer is transient and reversible, it has been widely observed across multiple CAR-T cell products and is implicated in long-term tumor recurrence [93]. Given the challenges of endogenous target antigen expression and ubiquitous trogocytosis-mediated antigen transfer, this phenomenon demands particular attention in in vivo CAR-T applications.

With advances in mechanistic research and engineering technology, several approaches have been proposed to mitigate fratricide induced by trogocytosis. It has been established that activation of transcription factor 3 downregulation of cholesterol-25-hydroxylase, driven by tumor-derived factors, serves as a key regulatory mechanism for trogocytosis in CAR immune cell therapy [94]. Therefore, targeting ATF3 and CH25H, such as downregulating ATF3 or upregulating CH25H can modulate trogocytosis. Methods for dynamically regulating CAR molecule expression can directly reduce the occurrence of trogocytosis [95]. Moreover, compared to high-affinity CAR-T cells, low-affinity CAR-T cells exhibit significantly less trogocytosis while maintaining potent anti-tumor activity. In contrast, high-affinity CAR-T cells undergo increased apoptosis and show reduced expansion capacity, indicating that trogocytosis can be mitigated by adjusting the affinity of CAR molecules [96].

### Potential challenges in the clinical application of in vivo CAR-T

#### Persistence

The limited durability of therapeutic effects remains a major challenge in adoptive CAR-T cell therapy across multiple diseases and is considered a primary cause of antigen-positive relapse [97]. This limitation is associated with CAR construct design, ex vivo manipulation, lymphodepletion conditioning, and T-cell exhaustion [98]. While in vivo CAR-T manufacturing circumvents ex vivo manipulation and lymphodepletion, persistence-related challenges persist.

As previously discussed, key parameters of CAR molecular structure significantly impact persistence. The affinity of the extracellular antigen-binding domain directly affects tonic signaling, where excessive affinity may drive T-cell exhaustion [99]. The costimulatory domain also critically influences CAR-T cell persistence and efficacy: CD28 intracellular domains promote cytokine production, whereas 4-1BB enhances long-term persistence [100]. Additionally, although mechanisms remain incompletely understood, hinge and transmembrane domains can modulate overall CAR-T performance, including in vivo persistence [101].

A more formidable challenge lies in T-cell exhaustion induced by persistent antigen stimulation and the immunosuppressive TME. This exhausted state manifests through multiple phenotypic alterations, including: loss of proliferative capacity, diminished cytokine production, impaired cytotoxic effector function, metabolic reprogramming (e.g., shifted from oxidative phosphorylation to glycolysis), characteristic transcriptional and epigenetic remodeling [102]. Furthermore, trogocytosis-mediated bidirectional transfer of CAR molecules and target antigens contributes to CAR-T cell exhaustion and is implicated in clinical relapse post CAR-T therapy [93]. Given that in vivo CAR-T manufacturing relies on endogenous patient T-cells, with limited options to augment cell numbers or refresh the CAR-T population, exhaustion may represent a critical determinant of therapeutic success. Notably, the absence of lymphodepletion in in vivo CAR-T approaches preserves host immune clearance of delivery vectors, potentially further limiting persistence [103].

#### Serious adverse reactions

The remarkable target cell eradication capacity of CAR-T cells comes with a consequential risk of excessive immune activation, primarily characterized by hyperproduction of pro-inflammatory cytokines (such as IL-6, IFN-γ, and IL-2) by both CAR-T cells and downstream myeloid cells. This cytokine storm can trigger potentially life-threatening complications, including CRS and immune effector cell-associated neurotoxicity syndrome (ICANS) [104]. In clinical studies of in vivo CAR-T therapy, patients often experience low-grade CRS and hematologic toxicities that can be effectively controlled.

Now, IL-6 receptor antagonist and glucocorticoid are commonly used to manage the related adverse reactions, so that the safety of CAR-T therapy can be guaranteed [105]. Furthermore, several innovative strategies are being explored to improve its safety. Among these, logic-gate systems based on suicide genes or small molecule-controlled switches represent promising approaches. These designs enable external intervention in response to patient-specific reactions, allowing precise spatial and temporal control over CAR-T cell activity [106]. A classic example is the inducible caspase 9 suicide switch, which can be activated by a specific small molecule to rapidly eliminate CAR-T cells via apoptosis [107]. Alternatively, a reverse CAR system decouples antigen recognition from T cell activation, relying instead on bispecific targeting molecules to redirect cytotoxicity flexibly against multiple antigens. This system incorporates “OR” or “AND” logic gates to enhance safety [108]. Another innovative approach, the inhibitory CAR (iCAR), incorporates inhibitory signaling domains. Upon recognition of off-tumor antigens, iCAR suppresses T cell activation, thereby mitigating on-target, off-tumor toxicity [109].

While these interventions have substantially improved the safety profile of conventional adoptive CAR-T therapy, the unique pharmacological context of in vivo CAR-T approaches necessitates a thorough reevaluation of the kinetics and magnitude of immune-related adverse events, potential modifications to existing toxicity management protocols, and the risk-benefit ratio in different patient populations. Particular attention should be paid to the possibility of more rapid cytokine surge due to decentralized CAR-T cell activation, potential differences in neurotoxicity profiles, long-term immunological consequences of in vivo CAR-T generation [110]. This reassessment is crucial for establishing safe clinical translation of in vivo CAR-T platforms.

Furthermore, secondary toxicities including long-term cytopenias, infections, and secondary malignancies require careful consideration. Infection-related mortality represents a predominant cause of CAR-T therapy-associated non-relapse death, exhibiting distinct temporal patterns [111]. Clinical evidence indicates that bacterial infections predominate within the first 30 days post-treatment, potentially associated with neutropenia induced by lymphodepleting chemotherapy [111]. Subsequently, viral infections, particularly those caused by herpesviruses and enteroviruses, become more prevalent [112]. While in vivo CAR-T therapy obviates the need for lymphodepletion and reduces early infection risk, vigilant management of CRS remains crucial. Related research has demonstrated that severe CRS correlates significantly with increased risk of late-onset infections [113].

The risk of secondary malignancies following CAR-T therapy (including both CAR-positive and CAR-negative tumors) has recently garnered significant attention. Although rare (incidence: 0.12%–0.17%), these cases have raised concerns regarding the long-term safety of CAR-T therapy [114]. Miklos et al. analyzed 724 patients who underwent cellular therapy at the Stanford Cancer Institute, identifying 25 cases of secondary malignancies [114]. Ghilardi et al. conducted a retrospective analysis of 449 CAR-T-treated patients at the University of Pennsylvania Medical Center, detecting 16 secondary malignancies, with a projected 5 year incidence of 17.0% [115]. Umyarova et al. evaluated 246 patients with relapsed/refractory B-cell lymphoma or multiple myeloma treated with CAR-T between 2016 and 2022, finding that 8.5% developed secondary primary malignancies, including non-melanoma skin cancer (52%), hematologic malignancies (33%), and non-cutaneous solid tumors (14%) [116]. These data identify secondary primary malignancies (SPMs) following CAR-T therapy as a novel clinically relevant long-term adverse event of CAR-T treatment. However, the benefits of CAR-T therapy remain evident, and studies have shown that the frequency of SPMs with CAR-T therapy is not higher than that observed with previous standard-of-care strategies [117].

At present, the cause of this secondary tumor has not been fully studied and clarified. Researchers speculate that this is related to the extremely weak risk signal associated with CAR-T therapy itself, the long-term complex interaction between CAR-T cells and host cells, and the risk of secondary malignancy already existing in the tumor patient population, such as the risk of malignant transformation caused by the damage of cellular genetic material in the body caused by the patient’s previous treatment [117, 118]. For in vivo CAR-T platforms, as the absence of ex vivo manufacturing precludes rigorous CAR-T cell characterization. Therefore, additional considerations include viral vector-mediated genotoxicity (e.g., insertional mutagenesis due to random integration), and off-target CAR expression. At the same time, it is necessary to strengthen the monitoring of patients who received CAR-T treatment after extensive treatment history, and establish a long-term monitoring mechanism for all patients who received CAR-T treatment [119].

## Potential enhancement strategies based on gene editing: insights from adoptive CAR-T

How to enhance the efficacy of CAR-T adoptive therapy is a vast and complex proposition, with the current primary research focus being on solid tumor treatment. From the perspective of the interaction between CAR-T cells and tumor cells, efforts are needed on two fronts. On one hand, it is necessary to enhance the effect of CAR-T on tumors, including their cytotoxic effects, the activation of systemic anti-tumor immunity, and the maintenance of long-term therapeutic outcomes [120]. On the other hand, it is essential to mitigate the tumor’s inhibitory mechanisms against CAR-T cells. Typical inhibitory factors include physical barriers, the immunosuppressive TME, repeated antigen stimulation, immune evasion, and metabolic interference (such as nutrient deprivation and hypoxia) [121]. The combined effects of these adverse factors may lead to CAR-T therapy failure or long-term relapse.

**Fig. 6:**
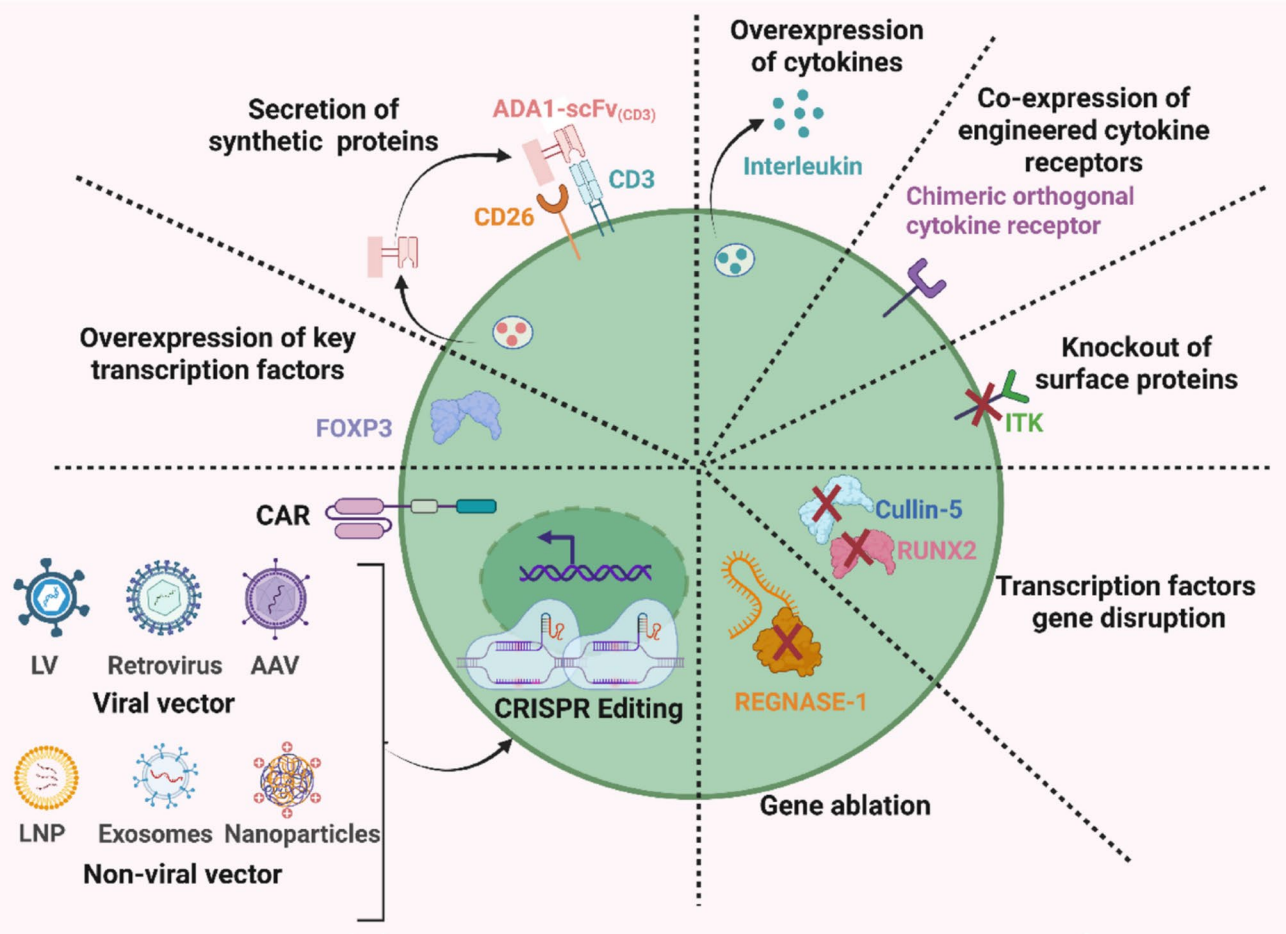
The combined application of CAR gene transduction and CRISPR-Cas9 genome editing represents a potential enhancement strategy for in vivo CAR-T therapy. Using the CRISPR-Cas9 system, targeted genes (such as transcription factors, intracellular enzymes, surface proteins, cytokines, engineered synthetic proteins) knock-in or knockout can be performed simultaneously with CAR gene delivery to enhance the functionality, persistence, and efficacy of in vivo CAR-T cells. *LV* Lentivirus, *AAV* Adeno-associated virus, *LNP* Lipid nanoparticle

Numerous engineering strategies have already been developed to enhance or improve the functionality of adoptive CAR-T cells. These include Co-expression of cytokines/chemokines or modification of their receptors, knocking out or over-expressing key regulatory genes, combining with immune checkpoint inhibitors, chemotherapy, radiotherapy, or other therapeutic approaches, and optimizing CAR structural design [122]. Given the mechanistic consistency between in vivo CAR-T and adoptive therapy, some gene-editing-based methods (such as CRISPR-Cas9) may be employed to enhance in vivo CAR-T functionality and performance (Fig. 6**)**. It is noteworthy that the inherent off-target risks associated with CRISPR-Cas9-based genome editing, combined with the potential non-specific tissue targeting of in vivo delivery vectors, may pose a synergistic risk. Specifically, off-target editing events occurring in non-intended tissues could lead to consequences that are difficult to predict and monitor. To address this issue, the use of self-inactivating or temporally controllable Cas9 systems, limiting their activity to a defined time window, may help reduce the likelihood of off-target damage [123]. Additionally, the development of novel delivery vectors with enhanced tissue and cell-type specificity should be incorporated into future strategies.

### Metabolic reprogramming of CAR-T

Numerous studies have demonstrated that enhancing the metabolic fitness of T-cells can improve their anti-tumor activity and persistence [124]. Given the close relationship between CAR-T cell metabolism and function, researchers are actively exploring strategies to optimize CAR-T metabolic states to overcome current limitations [125]. Treg cells exhibit a unique metabolic profile that enables their long-term survival and robust expansion within the hypoxic and acidic TME, with FOXP3 serving as a key metabolic regulator [126]. Niu et al. proposed and validated an intriguing approach: selectively harnessing FOXP3’s metabolic regulatory function to enhance CAR-T activity without conferring immunosuppressive properties. Their study revealed that CAR-T_Foxp3_ cells underwent significant metabolic reprogramming, characterized by reduced aerobic glycolysis and oxidative phosphorylation alongside elevated lipid metabolism. This modification conferred potent in vivo anti-tumor efficacy without inducing immunosuppression [127].

Adenosine, an immunosuppressive nucleoside, has been implicated in CAR-T dysfunction, as exhausted CAR-T cells up-regulate CD39 and CD73 to produce adenosine, thereby mediating immune suppression [128]. However, adenosine can be metabolized by adenosine deaminase (ADA) into inosine, a potent inducer of T-cell stemness that modulates T-cell metabolism, enhances functionality, and drives epigenetic reprogramming [129]. Crystal L Mackall’s team demonstrated that direct supplementation or over-expression of ADA depletes adenosine while increasing inosine levels, thereby improving CAR-T cell function and stemness [130]. Similarly, Hu et al. developed a metabolic fueling strategy for CAR-T cells using inosine as an alternative energy source [131]. ADA1, an isoform of ADA, is secreted via a non-classical pathway and present in human plasma, where it exerts co-stimulatory effects on T-cell-mediated immunity through CD26 binding. By co-expressing CD26 and secreting ADA1-CD3ζ in CAR-T cells, the researchers engineered a system where CD26 captures ADA1 in a membrane-proximal manner, enhancing CAR-T proliferation, cytokine production, and resistance to TGF-β1 suppression.

As early as 2019, Wei et al. employed CRISPR-Cas9 screening of metabolic regulators and identified REGNASE-1 as a major negative regulator of CAR-T anti-tumor responses in solid tumors [132]. REGNASE-1-deficient CD8^+^ T-cells were reprogrammed into long-lived effector cells with enhanced BATF activity and mitochondrial metabolism, leading to improved adoptive therapy outcomes. Similarly, studies on hematologic malignancies have demonstrated that REGNASE-1 directly targets TCF7 mRNA, and its deficiency promotes TCF-1 expression, thereby enhancing the persistence of anti-tumor response of CAR-T cells [133]. These findings suggest that REGNASE-1 knockout represents a broadly applicable strategy for CAR-T therapy, with potential utility in in vivo CAR-T development.

### Signal pathway-enhanced CAR-T

Recent advances in genome-wide screening approaches have identified key signaling modulators that can be targeted to enhance CAR-T cell efficacy. Adachi et al. performed a genome-wide CRISPR screen to identify genes regulating CAR-T effector function and discovered Cullin-5 as a critical determinant of CAR-T survival [134]. Genetic ablation of Cullin-5 augmented CAR-T proliferation and effector activity through upregulation of the JAK/STAT signaling pathway, leading to improved therapeutic outcomes in aggressive cancers. This study highlights the ubiquitin system as a promising target for enhancing CAR-T cell immunotherapy and provides a rational design strategy for next-generation CAR constructs.

Further investigations into TCR signaling revealed IL-2-inducible T-cell kinase (ITK) as a pivotal regulator of T-cell activation and differentiation. Fu et al. demonstrated that ITK knockout or inhibition mitigated CAR-T cell exhaustion while promoting a memory-like phenotype, resulting in prolonged persistence and sustained anti-tumor activity in vivo [135]. These findings suggest that ITK-deficient CAR-T cells represent a potential long-term therapeutic option.

In a separate study, Terry J Fry’s team explored the impact of antigen experience history on CD8^+^ CAR-T cell responses in leukemia [136]. They identified RUNX2, a transcription factor, as a key regulator of CAR-T cell differentiation. RUNX2 over-expression was shown to reduce exhaustion while preserving memory-like properties, ultimately enhancing anti-tumor potency. This work underscores the potential of transcriptional reprogramming to improve CAR-T cell durability and function.

### Cytokine-enhanced CAR-T

Beyond key regulatory genes, leveraging cytokine signaling through co-expression of cytokines or engineered cytokine receptors has emerged as a promising strategy to enhance CAR-T cell activation, expansion, persistence, and resistance to the TME. Supported by extensive pre-clinical and clinical evidence, this approach has been established as a fourth-generation CAR-T therapy [137].

IL-18 is the first cytokine-enhanced CAR-T therapy to achieve clear clinical results in lymphoma. Patients who relapsed after prior CD19 CAR-T treatment achieved an ORR of 81% and CR rate of 52%. All dose levels showed stable CAR-T cell expansion without unexpected adverse events [138]. Notably, unlike common γ-chain interleukins or other cytokines, endogenous IL-18 binding protein (IL18BP) naturally regulates IL-18 levels in humans [139]. This unique regulatory mechanism maintains safe IL-18 concentrations, favoring localized rather than systemic activity of IL-18-secreting CD19 CAR-T cells. In addition, conditionally activated IL-18 CAR-T products have also been designed. Hull et al. developed a granzyme B activated IL-18 CAR-T cell, which achieves safe control of CAR-T through the mechanism of release of specific molecules after activation of this T-cell [140].

In addition to IL-18, cytokine secreting CAR-T cells such as IL-7, IL-12, IL-15, and IL-21 have also entered the clinical trial phase and shown positive clinical outcomes [141, 142]. However, unlike IL-18, the continued secretion of these cytokines raises safety concerns. Several methods have been proposed to address this issue, such as Allen et al. designing a CAR-T cell carrying a synthetic cytokine circuit that drives IL-2 production through tumor specific synthetic Notch receptors, promoting CAR-T cell infiltration into solid tumors and producing local effects [143]. The orthogonal cytokine system has also been developed to alleviate excessive secretion of cytokines [144, 145]. This method involves mutating cytokine receptors to only respond to specific cytokine variants and not interact with wild-type cytokines, thereby avoiding immune system overreaction [146]. Indeed, in vivo CAR-T co-expressing IL-7 has been tested in animal models and successfully eliminated tumors, suggesting the applicability of cytokine-based enhancement strategies for in vivo CAR-T therapy.

### Potential combination therapy

Combination strategies represent a promising approach to enhance long-term remission in hematological malignancies and improve efficacy against solid tumors following CAR-T therapy, thereby broadening its therapeutic applicability and safety profile [122]. The combination of CAR-T adoptive therapy with immune checkpoint inhibitors has garnered support from multiple clinical studies across various cancers. For example, in hematologic malignancies, PD-1 blockade has been shown to reverse T cell exhaustion after anti-CD19 CAR-T treatment, leading to improved clinical outcomes in relapsed or refractory patients [147]. Similarly, in multiple myeloma, PD-1 inhibitors may restore T cell function, thereby reactivating the anti-tumor activity of CAR-T cells [148]. In certain solid tumors, such as glioma and malignant pleural tumors, combined PD-1 inhibition and CAR-T therapy has demonstrated substantial anti-tumor responses with a favorable safety profile [149, 150]. Additionally, novel strategies aimed at enhancing CAR-T function through targeting alternative immune checkpoints, such as disrupting the CD200-CD200R axis or inhibiting phagocytic checkpoints on tumor-associated macrophages, are under investigation [151, 152].

Other combination strategies, such as radiotherapy, tumor vaccine, monoclonal antibodies, and small-molecule compounds combined with CAR-T adoptive therapy, have also entered clinical research stages and have shown promising results in improving efficacy and safety [122]. Among them, RNA vaccines have been shown to have synergistic effects with in vivo CAR immune cells [57]. Clearly, further research is required to evaluate the application value of these combination strategies in in vivo CAR engineering. Nevertheless, it is undeniable that they offer potential support for optimizing the efficacy of in vivo CAR-T therapy and treating refractory diseases in the future.

## Advances in clinical studies of in vivo CAR-T therapy

Pre-clinical studies of in vivo CAR-T therapy face significant challenges in replicating the complex disease microenvironment of humans. Although patient-derived xenograft models have gradually replaced human tumor cell lines in cancer research, and immunodeficient mice have been upgraded to humanized models (such as Hu-PBMC and Hu-HSC), these systems still fail to accurately recapitulate the TME or the dynamic interactions between CAR-T cells and other immune components [153]. Consequently, the mechanisms underlying in vivo CAR-T generation and therapeutic efficacy remain poorly understood, impeding further progress in the field.

Furthermore, safety remains a critical concern at this stage. Unlike conventional CAR-T therapies, in vivo-generated CAR-T therapies require particular attention in aspects such as targeting delivery accuracy, persistence of CAR expression, immunogenic responses, and long-term effects due to their in vivo generation characteristics. However, real-time quantitative monitoring of in vivo CAR expression and controllable “safety switch” strategies are still under exploration. Additionally, although the FDA has lifted the mandatory risk evaluation and mitigation strategies requirements for autologous CAR-T therapies, in vivo-generated CAR-T, as an emerging technology, still necessitates the collection and evaluation of long-term safety and efficacy data, along with strict long-term safety follow-up requirements. The in vivo generation nature of these therapies also poses challenges for regulatory agencies, requiring exploration of how to better integrate such “off-the-shelf” drugs into existing regulatory frameworks while balancing innovation benefits and risk control [154].

Currently, in vivo CAR-T therapy has progressed to clinical trials (Table 3), with investigations underway for B-cell malignancies, leukemia, and autoimmune diseases. In anti-tumor applications, research has predominantly focused on well-validated targets such as CD19, CD20, and BCMA while in vivo CAR-T generation primarily employs lentiviral-based CAR gene delivery, with targeting strategies involving CD3, CD8, CD7, and anti-TCR nanobody. To date, only a limited number of studies have reported preliminary clinical outcomes for this emerging therapeutic strategy. ESO-T01 is a CAR lentiviral vector designed to mediate targeted T cell editing in vivo. It incorporates a humanized single-domain antibody targeting BCMA and utilizes a nanobody-based platform for T cell recognition. In a clinical evaluation involving four patients with BCMA-expressing refractory multiple myeloma, the treatment demonstrated promising efficacy. After two months of follow-up, two patients achieved complete remission, while the other two exhibited partial remission, accompanied by reduced tumor burden and minimal residual disease-negative status in the bone marrow. Although CRS and hematologic toxicity were commonly observed during treatment, all adverse events were manageable and remained under stable control [155]. JY231 has reported partial clinical data: a patient with relapsed/refractory diffuse large B-cell lymphoma and high tumor burden achieved complete remission and was discharged following JY231 treatment. Notably, the patient maintained stable vital signs and experienced no grade ≥ 2 CRS or neurotoxicity, which common adverse events associated with adoptive CAR-T therapy [156]. These cases underscores the therapeutic potential of in vivo CAR-T in oncology and expands the clinical feasibility of CAR-T manufacturing.

**Table 3:** Clinical trials of in vivo CAR-T therapy

Registration ID	Product	Phase	Indication	Targeted antigen	CAR gene vectors	Vector targeting	Project progress	Study start	Clinical sponsors	Country
NCT06539338	INT2104	Phase 1	Relapsed/refractory B-cell malignancies	CD20	Lentivirus	CD7	The patient was enrolled and received treatment	2024-10	Interius BioTherapeutics Inc.	Australia
NCT06689917	JY231	Not applicable	Relapsed/refractory B-cell lymphoma/leukemia	CD19	Lentivirus	CD3	A patient with relapsed/refractory diffuse large B-cell lymphoma achieved complete remission after treatment	2024-12	Tongji Hospital	China
NCT06691685	ESO-T01	Early Phase 1	Relapsed/refractory multiple myeloma	BCMA	Lentivirus	TCR	Of the four patients who received a single infusion of ESO-T01, two achieved complete remission and two achieved partial remission	2024-11	Union Hospital, Tongji Medical College	China
NCT06528301	UB-VV111	Phase 1	Relapsed/refractory large B-cell lymphoma and chronic lymphocytic leukemia	CD19	Lentivirus	CD3	Patient recruitment	2025-03	Umoja Biopharma	USA
NCT06618313	JCXH-213	Not applicable	Relapsed/refractory B-cell non-Hodgkin Lymphoma	CD19	LNP-mRNA	Not report	Patient recruitment	2025-03	Beijing GoBroad Hospital	China
NCT06917742	CPTX2309	Phase 1	Not applicable (healthy volunteer study)	CD20	LNP-mRNA	CD8	Patient recruitment	2025-04	Capstan Therapeutics	USA
NCT07065279	JY231	Not applicable	Relapsed/refractory B-cell malignancies	CD19	Lentivirus	CD3	Patient recruitment	2025-07	920th Hospital	China
NCT07059169	JY231	Not applicable	Refractory autoimmune diseases	CD19	Lentivirus	CD3	Not report	2025-07	Shenzhen Genocury Biotech Co., Ltd.	China
NCT06801119	HN2301	Not applicable	Systemic lupus erythematosus	CD19	LNP-mRNA	CD8	At 3 months after HN2301 treatment, all five patients showed improvement in their disease status	2025-03	Shenzhen MagicRNA Biotechnology Co., Ltd	China

Data were obtained from https://clinicaltrials.gov/

Additionally, in vivo CAR-T therapy is being explored for autoimmune diseases. An ongoing clinical trial (NCT06917742) is recruiting healthy volunteers to assess the safety and tolerability of CPTX2309, an mRNA-LNP-based in vivo CAR-T product targeting systemic lupus erythematosus (SLE) and sjogren’s syndrome. The latest clinical trial results support the application of in vivo CAR-T therapy in SLE. Five patients with refractory SLE were treated with HN2301, an engineered LNPs specifically designed to target CD8⁺ T cells. This nanoparticle contains encapsulated mRNA encoding anti-CD19 CAR, enabling direct editing of CAR-T cells within the patients’ bodies. CD8⁺ CD19 CAR-T cells were detected in peripheral blood as early as 6 h after infusion, with a low off-target expression rate of CAR. No severe adverse reactions were observed in any of the patients, indicating a favorable safety profile. The therapeutic effect was remarkable, with a sharp reduction in circulating B cell levels within 6 h after the first treatment, leading to complete depletion. By the 3-month follow-up, all five patients showed improvements in their disease condition [157].

In vivo CAR-T therapy not only offers considerable logistical advantages but also demonstrates potent B cell depletion in both pre-clinical and clinical research. These outcomes underscore its potential as a novel therapeutic strategy for autoimmune diseases, highlighting promising prospects for clinical translation.

## Conclusion and outlook

Since the approval of the first CAR-T cell therapy in 2017, this treatment has emerged as a cornerstone of cancer immunotherapy, particularly for hematologic malignancies. Beyond oncology, CAR-T therapy has demonstrated potential in managing refractory non-neoplastic conditions, including autoimmune diseases, fibrotic disorders, and chronic infections.

The expanding therapeutic indications for CAR-T therapies have intensified demand, yet manufacturing bottlenecks, supply constraints, and prohibitive costs hinder widespread adoption. Consequently, the field’s focus has shifted from therapeutic development to optimizing production scalability, affordability, and accessibility. As we discuss herein, in vivo CAR-T has the potential to be a “wall breaker” for this problem, drive the complete upgrade and widespread adoption of the cell therapy industry by providing ready-to-use, universal, and affordable CAR therapy products.

In summary, in vivo CAR-T generation represents a transformative alternative to conventional adoptive cell therapy, CAR-T cells are generated in vivo through viral vectors, nanomaterials and in vivo implantation platforms, circumventing key limitations such as ex vivo manufacturing complexity, prolonged production timelines, and high costs. This approach may also reduce preconditioning-associated risks, including lymphodepletion-induced infections and GVHD in allogeneic settings. Although challenges in technology and clinical application remain, including limited in vivo persistence, adverse effects, and long-term risks associated with CAR-T cell therapy, as well as unique obstacles related to in vivo CAR-T techniques, such as precise CAR gene delivery, editing efficiency, and controlled in vivo activity. However, benefiting from the research foundation and enlightenment of adoptive therapy, targeted programs and enhancement strategies have been proposed, and clinical research has continued to advance and achieved preliminary positive results. These advances underscore its potential to redefine CAR-T therapy, offering a scalable platform for broader clinical implementation in the near future.

## Data Availability

No datasets were generated or analysed during the current study.

## Abbreviations

AAV: Adeno-associated virus
ADA: Adenosine deaminase
CAR: Chimeric antigen receptor
CAR-M: CAR-Macrophage
CRS: Cytokine release syndrome
DCR: Disease control rate
EFS: Event-free survival
GVHD: Graft-versus-host disease
ICANS: Immune effector cell-associated neurotoxicity syndrome
IL-18BP: IL-18 binding protein
iPSC: induced pluripotent stem cell
ITAM: Immunoreceptor tyrosine-based activation motif
ITK: IL-2-inducible T-cell kinase
LNP: Lipid nanoparticle
MDF: Multi-domain fusion
ORR: Overall response rate
OS: Overall survival
PR: Partial response
PFS: Progression-free survival
RACR: Rapamycin-activated cytokine receptor
saRNA: Self-amplifying mRNA
SPMs: Secondary primary malignancies
SLE: Systemic lupus erythematosus
TCR: T-cell receptor
tLNP: Targeted lipid nanoparticle
